# Fc gamma receptor IIa suppresses type I and III interferon production by human myeloid immune cells

**DOI:** 10.1002/eji.201847615

**Published:** 2018-09-14

**Authors:** Melissa Newling, Willianne Hoepel, Lisa T.C. Vogelpoel, Marieke H. Heineke, Johan A. van Burgsteden, Esther W.M. Taanman‐Kueter, Dirk Eggink, Taco W. Kuijpers, Tim Beaumont, Marjolein van Egmond, Martien L. Kapsenberg, Dominique L.P. Baeten, Jeroen den Dunnen, Esther C. de Jong

**Affiliations:** ^1^ Amsterdam Rheumatology and Immunology Center location Academic Medical Center (AMC) Amsterdam The Netherlands; ^2^ Amsterdam UMC University of Amsterdam Department of Experimental Immunology Amsterdam The Netherlands; ^3^ Amsterdam UMC Vrije Universiteit Amsterdam Department of Molecular Cell Biology and Immunology Amsterdam The Netherlands; ^4^ Amsterdam UMC University of Amsterdam Department of Medical Microbiology Amsterdam The Netherlands; ^5^ Department of Blood Cell Research Sanquin Research and Landsteiner Laboratory Amsterdam The Netherlands; ^6^ Department of Pediatric Hematology Immunology and Infectious Disease Emma Children's Hospital AMC University of Amsterdam Amsterdam The Netherlands; ^7^ AIMM Therapeutics AMC Amsterdam The Netherlands; ^8^ Amsterdam UMC Vrije Universiteit Amsterdam Department of Surgery Amsterdam the Netherlands

**Keywords:** antigen‐presenting cell, Fc gamma receptor, human myeloid immune cells, IgG, type I interferon

## Abstract

Type I and type III interferons (IFNs) are fundamental for antiviral immunity, but prolonged expression is also detrimental to the host. Therefore, upon viral infection high levels of type I and III IFNs are followed by a strong and rapid decline. However, the mechanisms responsible for this suppression are still largely unknown. Here, we show that IgG opsonization of model viruses influenza and respiratory syncytial virus (RSV) strongly and selectively suppressed type I and III IFN production by various human antigen‐presenting cells. This suppression was induced by selective inhibition of TLR, RIG‐I‐like receptor, and STING‐dependent type I and III IFN gene transcription. Surprisingly, type I and III IFN suppression was mediated by Syk and PI3K independent inhibitory signaling via FcγRIIa, thereby identifying a novel non‐canonical FcγRIIa pathway in myeloid cells. Together, these results indicate that IgG opsonization of viruses functions as a novel negative feedback mechanism in humans, which may play a role in the selective suppression of type I and III IFN responses during the late‐phase of viral infections. In addition, activation of this pathway may be used as a tool to limit type I IFN‐associated pathology.

## Introduction

Type I IFNs are fundamental in the initial phase of antiviral immune responses. The type I IFN family comprises multiple IFN subtypes, of which IFN‐α (13 homologous subtypes) and IFN‐β are the best defined and most broadly expressed 1, 2, 3, 4. Type I IFNs have both autocrine and paracrine effects and are therefore able to induce an antiviral state in both virus‐infected and uninfected bystander cells 1, 5. This antiviral state is predominantly achieved via the induction of several hundreds of IFN‐stimulated genes (ISGs), which interfere with multiple stages of the antiviral replication cycle, through various mechanisms 3, 4. Almost all cells can produce type I IFNs, usually in response to stimulation of PRRs that recognize nucleic acids, including TLRs, RIG‐I‐like receptors (RLRs), and cytosolic DNA sensors (CDS) that signal through the signaling molecule Stimulator of Interferon Genes (STING) 3, 4, 6, 7. However, predominantly specialized APCs, such as DCs are potent inducers of type I IFNs 3, 5. Functionally related to the type I IFN family is the type III IFN family, which includes IFN‐λ1 (IL‐29), IFN‐λ2 (IL‐28A), IFN‐λ3 (IL‐28B), and IFN‐λ4. DCs are also the main producers of type III IFNs, which exert functions similar to type I IFNs, although their activity is more restricted since the expression of their receptor is limited to specific cell types such as epithelial cells and DCs themselves 4, 8.

Despite the important role for type I IFNs in the early response to viral infections, prolonged type I IFN production can have severe detrimental effects, such as tissue damage and exacerbation of disease 4, 9. In addition, prolonged type I IFN expression contributes to pathogenesis during viral infection by inhibition of T cell responses 4, 10, 11 and B cell responses 12, 13. Therefore, it is crucial that type I IFN responses are inhibited during the late phase of viral infections. Indeed, during the course of a viral infection, the levels of type I IFN are tightly controlled, with high levels in the first few days followed by a strong and rapid decline, initially in blood and subsequently in local tissues 14, 15. However, remarkably, the endogenous mechanisms of the human immune system that are responsible for this essential late‐phase inhibition of type I IFN are still largely unknown.

Since the time of suppression of type I IFNs during viral infections coincides with the emergence of adaptive immune responses, particularly in infected tissues 14, 15, we here investigated whether the presence of virus particles or proteins that are opsonized by IgG antibodies, which are only present during late‐phase (or secondary) infections, function as an environmental cue to suppress type I and III IFN responses.

## Results

### IgG opsonization of influenza or RSV suppresses type I and III IFN and ISG responses

**Figure 1:**
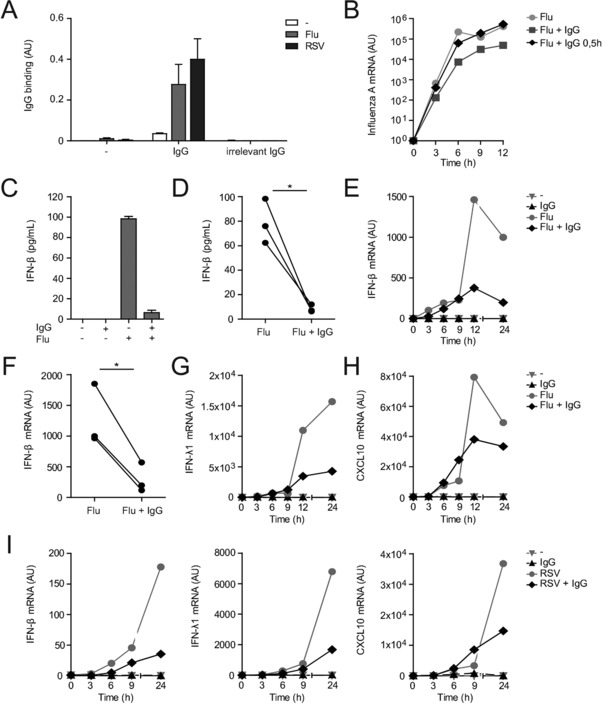
IgG opsonization of influenza or RSV suppresses type I and III IFN and ISG responses. (A) Influenza (Flu) or RSV were incubated with pooled IgG from healthy donors or irrelevant IgG. IgG binding to virus was determined by ELISA; mean ± SEM of triplicate is shown. (B) DCs were stimulated with influenza, either or not in the presence of IgG. IgG was added at the same time as influenza (Flu + IgG) or 0.5 h after addition of the virus (Flu + IgG, 0.5 h). (C–I) DCs were stimulated with influenza or RSV, either or not in the presence of IgG. (C and D) After 24 h, cytokine levels were determined by ELISA. (C) Data are shown as mean ± SEM of triplicate. (D) Each pair of dots represents one donor. ^*^ *p* < 0.05, paired two‐tailed Student's *t*‐test. (B, E–I) mRNA expression (at indicated time points) was determined by quantitative RT‐PCR. (F) mRNA levels were compared at *t* = 24 h. Each pair of dots represents one donor. *p < 0.05, paired two‐tailed Student's *t*‐test. (A–C, E, G–I) Data shown are from one experiment, representative of three independent experiments.

To study whether IgG opsonization affects cytokine production by myeloid APCs, the main cell type responsible for local type I and III IFN production in infected tissues 16, we made use of two frequently occurring model viruses, i.e., influenza (H3N2) and RSV (A2). Binding of pooled IgG to both viruses was verified as previously described using a binding ELISA 17 (Fig. 1A), thereby confirming that this pooled IgG could be used for opsonization of these viruses.

To assess the effect of IgG opsonization of viruses on IFN production by human myeloid APCs, we exposed human DCs to either non‐opsonized or IgG‐opsonized influenza and analyzed the production of type I and III IFNs. To exclude a potential role for antibodies in suppressing IFN production through virus neutralization (thus preventing infection and consequent infection‐related cytokine production), antiviral IgG antibodies were added 30 min after addition of the virus, thereby equalizing productive infection (Fig. 1B). Since IFN‐α is hardly or not produced by human DCs 5, we focused on IFN‐β as the main type I IFN. Strikingly, IgG opsonization of influenza strongly suppressed the production of IFN‐β (representative example in Fig. 1C, multiple donors in Fig. 1D). These data indicate that IgG opsonization of influenza inhibits type I IFN production by human DCs.

To determine whether the inhibition of IFN‐β production was mediated at the level of transcription, we determined IFN‐β mRNA expression over time using quantitative RT‐PCR. Analogous to protein levels, IgG opsonization of influenza strongly suppressed IFN‐β mRNA production (representative example in Fig. 1E, multiple donors in Fig. 1F). Similarly, IgG opsonization of influenza suppressed IFN‐λ1 mRNA expression (Fig. 1G).

One of the main functions of IFNs in orchestrating antiviral immunity is the induction of ISGs, such as the antiviral chemokine CXCL10 (also known as IFN‐γ‐induced protein 10; IP‐10) 18. Similar to IFN‐β and IFN‐λ1, IgG opsonization also suppressed influenza‐induced CXCL10 mRNA production (Fig. 1H).

In addition to influenza, we studied the effects of IgG opsonization of RSV. Similar to the effects we observed upon opsonization of influenza, IgG opsonization of RSV resulted in inhibition of IFN‐β, IFN‐λ1, and CXCL10 mRNA production (Fig. 1I). Taken together, these data indicate that IgG opsonization of viruses impairs type I and III IFN‐related immune responses by human DCs, which is regulated at the level of gene transcription.

### IgG immune complexes suppress TLR3, RIG‐I/MDA5, and CDS‐ induced type I and III IFN production

**Figure 2:**
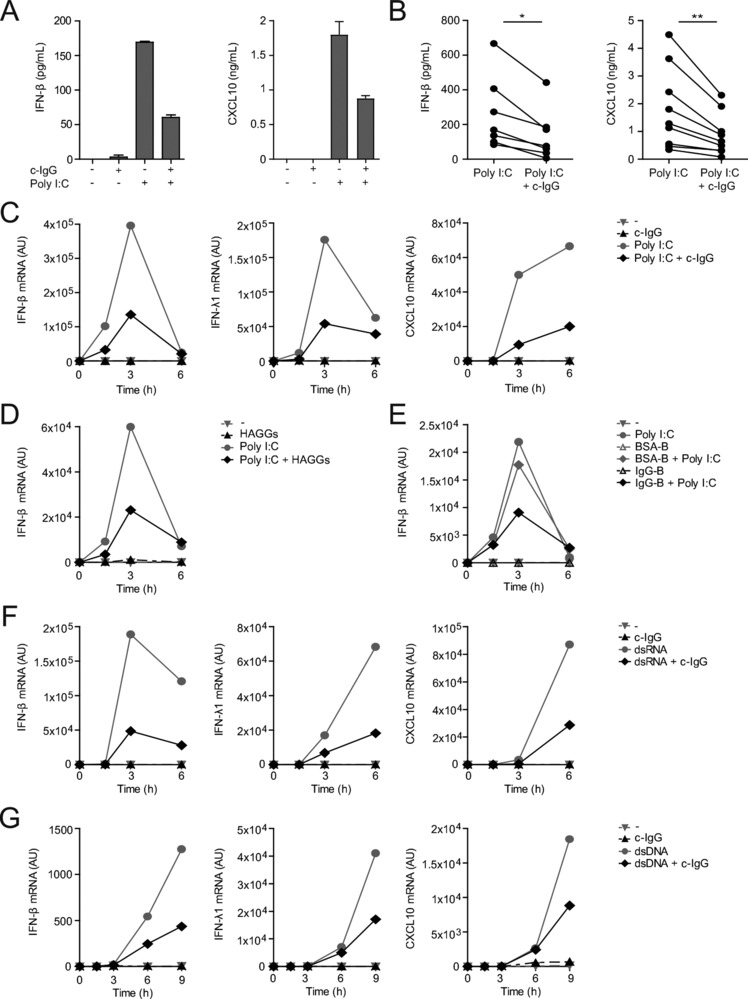
IgG immune complexes suppress type I and III IFN production induced by TLR3, RIG‐I/MDA5, and CDS. DCs were stimulated with TLR3 ligand Poly I:C (A–E), RIG‐I/MDA5 ligand Poly I:C (HMW)/LyoVec (F), or CDS ligand Poly (dG:dC)/LyoVec (G), either or not in combination with c‐IgG (A–C,F,G), HAGGs (D), or BSA‐B or IgG‐B (E). (A) After 3 h, cytokine levels were determined by ELISA, mean ± SEM of triplicate. (B) Each pair of dots represents one donor. *p < 0.05, **p < 0.01, paired two‐tailed Student's *t*‐test. (C–G) mRNA expression (at indicated time points) was determined by quantitative RT‐PCR. (C–G) Data shown are from one experiment, representative of 22 (C) or three (D–G) independent experiments.

Induction of type I and III IFNs by myeloid cells is predominantly mediated by activation of endosomal receptors such as TLRs, as well as cytosolic receptors for RNA (such as RIG‐I and MDA5) and DNA (such as cGAS and other STING signaling receptors) 4. To assess whether IgG suppresses IFN production induced by these families of virus‐sensing receptors, we stimulated DCs with specific ligands in combination with human complexed‐IgG (c‐IgG), generated by coating purified human IgG on high‐affinity plates. Individual stimulation of TLR3, which senses dsRNA that is expressed upon infection by most RNA viruses including influenza and RSV 6, 19, 20, 21, indeed induced IFN‐β and CXCL10 protein production (Fig. 2A). In contrast, stimulation with only c‐IgG did not induce IFN‐β or CXCL10 (Fig. 2A). However, combined stimulation with TLR3 ligand Poly I:C and c‐IgG, as occurs upon encountering IgG‐opsonized viruses, reduced IFN‐β and CXCL10 protein levels (representative example in Fig. 2A, multiple donors in Fig. 2B). Similarly, c‐IgG suppressed TLR3‐induced transcription of IFN‐β, IFN‐λ1, and CXCL10 (representative example in Fig. 2C, multiple donors in Supporting Information Fig. S1A). In addition, we assessed mRNA expression of IFN‐λ family members IFN‐λ2, IFN‐λ3, and IFN‐λ4. Similar to IFN‐λ1, co‐stimulation of Poly I:C with c‐IgG suppressed the transcription of IFN‐λ2 and IFN‐λ3 (Supporting Information Fig. S1B). IFN‐λ4 mRNA expression could not be detected in these cells.

Next, we ascertained that soluble immune complexes induce similar responses as plate‐bound IgG by two different approaches. First, we stimulated DCs with Poly I:C in combination with heat‐aggregated Igs (HAGGs) 22. Similar to plate‐bound IgG, co‐stimulation of Poly I:C with HAGGs suppressed the transcription of IFN‐β (Fig. 2D). Second, we co‐stimulated DCs with IgG‐coated beads (IgG‐B), for which we used BSA‐coated beads (BSA‐B) as a control. In line with previous findings, co‐stimulation with IgG‐B suppressed Poly I:C‐induced IFN‐β transcription, while BSA‐B did not (Fig. 2E). These data indicate that soluble and plate‐bound IgG immune complexes suppress type I IFN production by human DCs in a similar manner.

Next, we set out to investigate whether IgG immune complexes also affect type I and III IFN production induced by other virus‐sensing receptors. Similar to TLR3, c‐IgG also suppressed type I and III IFN responses induced by stimulation with cytosolic RNA and DNA, which activate RIG‐I/MDA5 and STING‐signaling receptors, respectively (Fig. 2F and G). Taken together, these data indicate that IgG immune complexes suppress type I and III IFN and ISG production in human DCs by a variety of virus‐sensing receptors.

### IgG immune complexes suppress type I and III IFN production in various human myeloid cells

**Figure 3:**
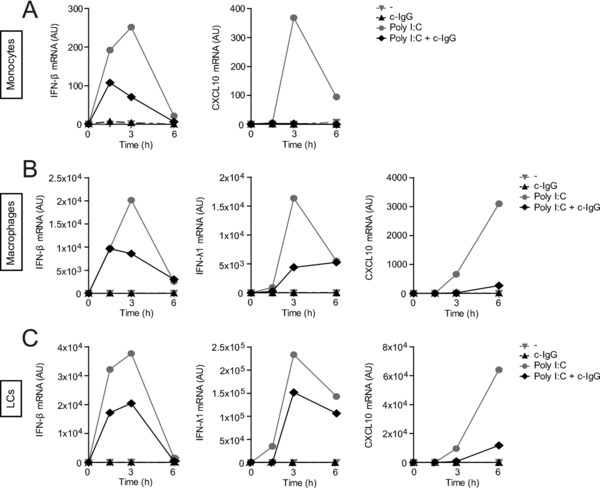
IgG immune complexes suppress type I and III IFN production in various human myeloid antigen‐presenting cells. Monocytes (A), macrophages (B), and LCs (C) were stimulated with Poly I:C, c‐IgG, or the combination. mRNA expression (at indicated time points) was determined by quantitative RT‐PCR. (A–C) Data shown are from one experiment, representative examples of three (A), or four (B–C) independent experiments.

To determine whether the suppression of type I and III IFN responses after IgG opsonization is specific for DCs or that it also holds true for other human myeloid APCs, we assessed the effect of co‐stimulation on monocytes, macrophages, and LCs. Similar to what we observed using DCs, combined stimulation of Poly I:C with c‐IgG also reduced the transcription of IFN‐β and CXCL10 in monocytes (Fig. 3A), macrophages (Fig. 3B), and LCs (Fig. 3C). Likewise, IFN‐λ1 mRNA expression was suppressed in macrophages and LCs (Fig. 3B and C, respectively), while IFN‐λ1 could not be detected in monocytes. These findings indicate that the suppression of type I and III IFNs by IgG immune complexes is functional in various cell types of the myeloid cell compartment.

### IgG immune complexes suppress IFN responses through FcγRIIa

**Figure 4:**
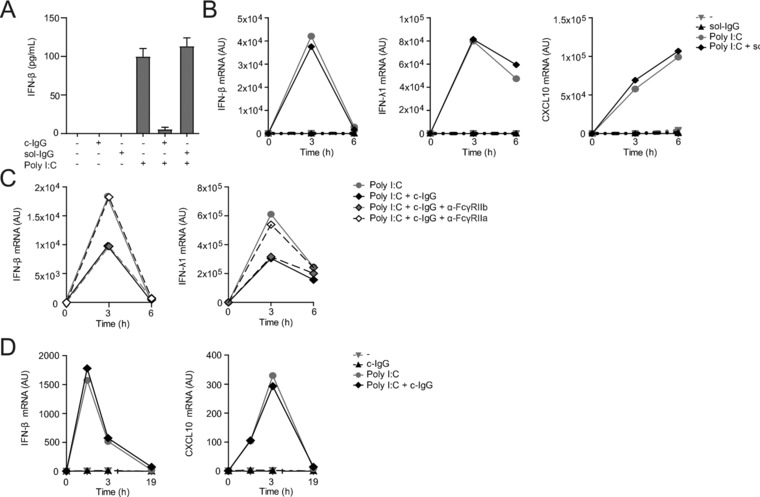
IgG‐mediated IFN suppression is dependent on FcγRIIa. Human DCs (A–C) and mouse DCs (D) were stimulated with Poly I:C, either or not combined with soluble IgG (sol‐IgG) (A and B) or c‐IgG (A, C, D), after pre‐incubation with an anti‐FcγRIIb or anti‐FcγRIIa blocking antibody (C). (A) After 3 h of stimulation, cytokine levels were determined by ELISA. Data are shown as mean ± SEM of triplicate. (B–D) mRNA expression (at indicated time points) was determined by quantitative RT‐PCR. (A–D) Data shown are from one experiment, representative of three independent experiments.

The main receptors on DCs for recognizing IgG immune complexes, as present on opsonized virus particles, are members of the FcγR family. Therefore, we hypothesized that the suppression of type I and III IFNs and ISGs upon IgG opsonization of viruses is the result of crosstalk between virus‐sensing receptors and FcγRs. Human myeloid cells express both high‐affinity receptor FcγRI (CD64), which recognizes soluble monomeric IgG, as well as low‐affinity receptors FcγRIIa (CD32a), FcγRIIb (CD32b), and FcγRIII (CD16), which can only recognize IgG after formation of IgG immune complexes 23. In contrast to stimulation with c‐IgG, stimulation with soluble IgG did not affect TLR3‐induced IFN‐β protein production (Fig. 4A) and IFN‐β, IFN‐λ1, or CXCL10 mRNA production (Fig. 4B). These data indicate that IgG complex formation is crucial for the inhibition of type I and III IFN and ISG responses. Since FcγRIIb is the only known classical ‘inhibitory’ FcγR, we blocked FcγRIIb using a specific antibody. However, surprisingly, blocking FcγRIIb had no effect on c‐IgG‐induced type I and III IFN suppression (Fig. 4C). In contrast, c‐IgG‐induced type I and III IFN suppression was completely counteracted by inhibition of FcγRIIa (Fig. 4C), which is generally considered to be an ‘activating’ receptor. These data identify FcγRIIa as the main receptor responsible for type I and III IFN and ISG suppression.

FcγRIIa is selectively expressed by primates and not by other species such as rodents 23. To test whether c‐IgG‐induced suppression is also functional in species such as mice (e.g., by alternative IgG‐binding receptors), we assessed IFN‐β and CXCL10 transcription by murine BM derived DCs. Strikingly, in contrast to human DCs, c‐IgG stimulation did not affect TLR3‐induced transcription of IFN‐β and CXCL10 by murine DCs (Fig. 4D). These data suggest that c‐IgG‐induced suppression of type I IFN responses may be restricted in mice. Collectively, these data demonstrate that IgG immune complex‐induced suppression of type I and III IFN production is dependent on FcγRIIa, which is functional in human but not murine myeloid cells.

### FcγRIIa co‐stimulation selectively suppresses type I and III IFN‐related genes, but not other antiviral genes

**Figure 5:**
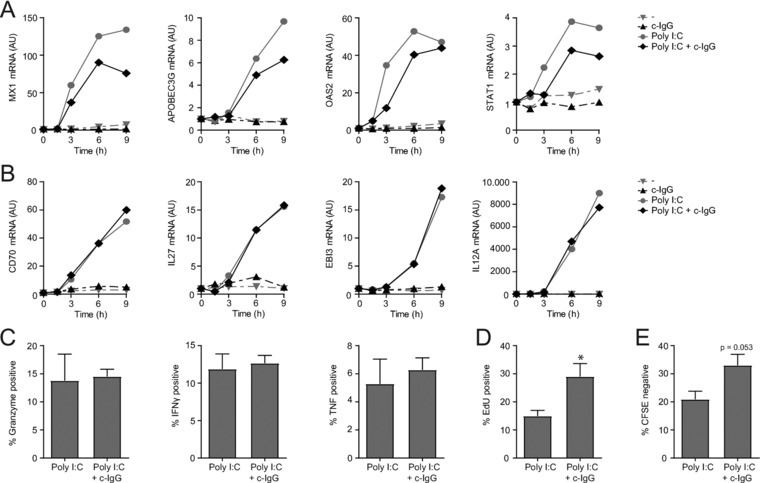
FcγRIIa co‐stimulation selectively suppresses type I and III IFNs. DCs were stimulated with Poly I:C, either or not combined with c‐IgG (A–E), and co‐cultured with allogeneic naïve CD8^+^ T cells (C–E). mRNA expression (at indicated time points) was determined by quantitative RT‐PCR. (A and B) Data shown are from one experiment, representative of three independent experiments. (C) Intracellular expression of granzyme B, IFN‐γ, or TNF was determined by flow cytometry. CD8^+^ T cell proliferation was determined by EdU incorporation (D) or CFSE labeling (E). (C–E) Data are pooled from three independent experiments, mean ± SEM. ^*^ *p *< 0.05, paired two‐tailed Student's *t*‐test.

Myeloid cells do not only counteract viral infections by production of type I and III IFNs, but also induce antiviral immunity through upregulation of various other proteins, such as cytokines and cell‐surface proteins that promote T cell activation. To determine which genes are affected by FcγRIIa co‐stimulation of myeloid cells, we assessed the transcription of a larger panel of antiviral genes. Following the reduced production of type I and III IFNs, stimulation with c‐IgG attenuated TLR3‐induced mRNA expression of ISGs MX1, APOBEC3G, OAS2, and STAT1 (Fig. 5A). In contrast, co‐stimulation with c‐IgG did not affect transcription of antiviral genes that are not dependent on autocrine type I IFN, such as CD70, IL‐27 subunits IL27 (encoding IL‐27p28) and EBI3, and rate‐limiting IL‐12 subunit IL12A (encoding IL‐12p35) (Fig. 5B). Since CD70, IL‐27, and IL‐12 are antiviral proteins that are crucial for T cell activation 24, 25, 26, 27, 28, these data suggest that FcγRIIa co‐stimulation of myeloid cells does not affect subsequent T cell activation. To test this, we co‐cultured DCs with CD8^+^ T cells and assessed T cell activation and proliferation. Indeed, stimulation of DCs with c‐IgG did not affect granzyme B, IFN‐γ, and TNF production by CD8^+^ T cells (Fig. 5C). Interestingly, CD8^+^ T cell proliferation, as determined by EdU incorporation (Fig. 5D) or CFSE labeling (Fig. 5E), was even increased upon co‐stimulation of DCs with c‐IgG. Hence, FcγRIIa‐TLR crosstalk selectively affects type I and III IFN responses, while it does not impair the induction of antiviral CD8^+^ T cell responses by human DCs.

### Inhibitory signaling by FcγRIIa is induced via a Syk‐ and PI3K‐independent pathway

**Figure 6:**
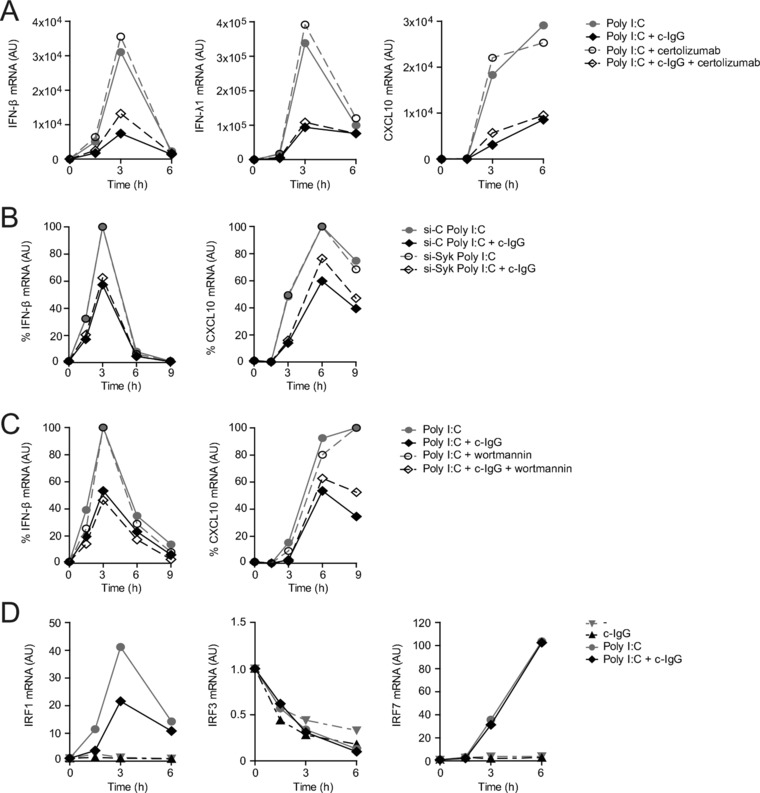
FcγRIIa‐mediated IFN suppression is Syk‐ and PI3K‐independent. DCs were stimulated with Poly I:C, either or not combined with c‐IgG and certolizumab (A). Syk was silenced using specific si‐RNA. Syk‐silenced DCs were stimulated with Poly I:C alone or combined with c‐IgG (B). DCs were pretreated with PI3K inhibitor wortmannin or a corresponding volume of DMSO and stimulated with Poly I:C or Poly I:C combined with c‐IgG (C). DCs were stimulated with Poly I:C, either or not combined with c‐IgG, after which mRNA expression of IRF1, IRF3, and IRF7 was determined (D). mRNA expression (at indicated time points) was determined by quantitative RT‐PCR. Data shown are from one experiment, representative of three (A and D), six (B), or four (C) independent experiments.

Subsequently, we set out to investigate the underlying molecular mechanisms of FcγRIIa‐induced suppression of type I and III IFNs. Previously, cross‐regulation between type I IFN and TNF has been proposed, in which both cytokines counteract each other 29. Indeed, after 3 and 6 h of stimulation with Poly I:C, we could already detect TNF protein levels, which were moderately increased at protein and mRNA level upon co‐stimulation with c‐IgG (Supporting Information Fig. S2A and B). To assess whether this elevated TNF is responsible for type I IFN suppression, we blocked TNF using anti‐TNF antibody certolizumab, which lacks an Fc tail and therefore does not interfere with FcγR activation 30, 31. However, as shown in Figure 6A, TNF inhibition had no effect on c‐IgG‐induced suppression of IFN‐β, IFN‐λ1, or CXCL10 transcription, indicating the involvement of other mechanisms.

Almost all previously described FcγRIIa‐mediated functions, including phagocytosis, degranulation, and pro‐inflammatory cytokine induction, are dependent on signaling via kinases Syk and PI3K 32, 33, 34. Therefore, we set out to determine whether FcγRIIa‐induced inhibition of type I and III IFN is also dependent on these upstream kinases. To assess the role of Syk, we silenced Syk expression using RNA interference (Fig. S3A). To verify functional Syk silencing, we assessed Syk‐dependent IL‐1β production as induced by TLR‐FcγR cross‐talk 17, 35, which was indeed fully blocked (Fig. S3B). However, strikingly, Syk silencing did not affect c‐IgG‐induced suppression of IFN‐β and CXCL10 transcription (Fig. 6B). In addition, inhibition of PI3K using three different inhibitors, i.e., wortmannin (Fig. 6C), LY294002 (Supporting Information Fig. S4A), and idelalisib (Supporting Information Fig. S4B), also did not affect the suppression of IFN‐β transcription.

Next, since FcγRIIa inhibits type I and III IFN production at the level of gene transcription, we assessed the expression of the key transcription factors of type I IFNs, i.e., IRF1, IRF3, and IRF7. Notably, co‐stimulation of Poly I:C with c‐IgG did not affect mRNA expression of IRF3 and IRF7, but did suppress mRNA expression of IRF1 (Fig. 6D). Taken together, these data indicate that inhibitory signaling by FcγRIIa is induced by a pathway that, in contrast to all other FcγRIIa‐mediated functions in myeloid cells, is independent of Syk and PI3K, and which leads to decreased expression of type I IFN transcription factor IRF1.

## Discussion

While it has long been known that type I IFNs are tightly controlled during viral infection, the responsible endogenous negative feedback mechanisms in humans are still largely unclear. Here, we have identified IgG opsonization of viruses as a novel mechanism that suppresses type I and III IFN production by human myeloid APCs. This suppression is induced by selective inhibition of type I and III IFN gene transcription. Importantly, type I and III IFN suppression is induced by inhibitory signaling via FcγRIIa that is independent of Syk and PI3K, indicative of a novel non‐canonical pathway of FcγRIIa signaling in myeloid cells. Together, these results indicate that IgG opsonization of viruses may function as an environmental cue to the human immune system to suppress type I and III IFN responses to prevent prolonged activation and concomitant pathology (for model see graphical abstract).

Our data demonstrate that FcγRIIa‐induced type I and III IFN suppression is functional in various myeloid cells, including DCs, LCs, macrophages, and monocytes. Located at the body's barriers such as skin and mucosal surfaces, these myeloid tissue APCs are the key cell types responsible for the local production of type I and III IFN during infection with viruses such as influenza 16. FcγRIIa suppresses type I and III IFN responses induced by different families of virus‐sensing receptors, including endosomal receptors such as TLRs and cytosolic receptors such as RLRs and CDS. Given this diversity in cell types as well as the families of receptors involved, this mechanism of type I and III IFN suppression is very likely to play a role during the majority of viral infections.

While FcγRIIa locally suppresses type I and III IFN production by myeloid APCs at the place of infection, FcγRIIa is also expressed by plasmacytoid DCs (pDCs), which mainly reside in blood and peripheral lymphoid organs and largely control systemic type I IFN production 36. However, previous reports indicate that on pDCs FcγRIIa co‐ligation promotes rather than inhibits type I IFN production 37, 38, 39, suggesting that this FcγRIIa‐dependent negative feedback mechanism may be specific for myeloid APCs. The reason for this cell‐type dependent difference is still speculative, but could be related to the fact that the effect of FcγRIIa activation on type I IFN production by pDCs has mainly been assessed in the context of chronic inflammatory disorders such as systemic lupus erythematosus (SLE) 37, 38, 39, which may differ from FcγRIIa activation in the context of viral infections. In addition, this difference could be related to the type of type I IFN involved, since pDCs mainly produce IFN‐α, instead of IFN‐β 14. Since IFN‐α is also systemically suppressed during late‐phase viral infection, it is very likely that pDCs bear a negative feedback mechanism for type I IFN production as well, although this may use a cue other than the presence of IgG immune complexes, e.g., signals that promote pDC apoptosis 40. In addition to the distinction between conventional DCs and pDCs, it is also important to realize that (particularly within the group of conventional DCs) various different DC subsets exist, which differ between, and even within, tissues 41. Since these subsets have different functions and characteristics, also the degree of suppression of type I IFNs by IgG immune complexes could differ. As such, this variation between DC subsets could contribute to the generation of tissue‐specific antiviral immunity, which is in line with previous findings that identified tissue‐specific cytokine responses induced by different FcR family members 23, 42.

Importantly, FcγRIIa co‐stimulation did not suppress antiviral genes in general, but specifically suppressed type I and III IFNs and related ISGs without impairing the expression of other antiviral genes. In addition, FcγRIIa co‐stimulation on myeloid cells increased CD8^+^ T cell proliferation. As such, FcγRIIa co‐stimulation of myeloid cells appears to actively promote the kinetics as observed during late‐phase viral infections, in which type I IFN rapidly decreases while T cell activation is increased 14. Recently, it was shown that in the context of HIV infection passively transferred antibodies promote CD8^+^ T cell responses, which was postulated to be induced by formation of immune complexes that activate FcRs on APCs 43. Importantly, our data provide evidence that supports this hypothesis, and suggests that this promotion of protective CD8^+^ T cell responses is dependent on FcγRIIa stimulation of myeloid APCs.

In contrast to human cells, stimulation with IgG immune complexes did not suppress type I IFN production by murine DCs. Several different explanations may account for this difference. First, in this study, we have used different DC differentiation protocols for human and mouse, i.e., differentiation of monocytes from blood using GM‐CSF + IL‐4 for human DCs, and BM differentiated with GM‐CSF for murine DCs. While these protocols are the most commonly used differentiation protocols for human and mouse, these distinct protocols may account for the observed differences in FcR‐induced IFN‐β suppression. Alternatively, the difference between human and mouse may be explained by the difference in expression of FcγRIIa, which was the main receptor responsible for type I and III IFN suppression by human myeloid cells, but has no direct homolog in mice 23. However, since type I IFN responses are also restricted in late‐phase and secondary viral infections in mice 44, 45, it seems likely that rodents have developed alternative mechanisms for late‐phase type I IFN suppression. Indeed, it has recently been shown that IgG opsonization of viruses prevents type I IFN production by murine pDCs via FcγRIIb, which prevents cargo from accessing TLR signaling endosomes 46. Interestingly, this suppression was reversed by transgenic expression of human FcγRIIa 46, which may explain the lack of FcγRIIb‐dependent suppression by IgG immune complexes in pDCs of humans. In contrast to pDCs, it is still unclear how type I IFN responses are inhibited by myeloid cells of mice, but our data indicate it may not involve IgG immune complex formation.

Surprisingly, suppression of type I and III IFNs by IgG opsonization of viruses was dependent on the classic ‘activating’ FcR FcγRIIa, and not FcγRIIb, which is the only ‘inhibitory’ FcR. As such, these findings mark the identification of a novel inhibitory function of FcγRIIa. Notably, negative control of inflammatory responses has been described before for FcγRIIa, a process which has been denoted inhibitory ITAM (ITAMi) signaling 47, 48, 49. However, ITAMi signaling is unlikely to be responsible for type I and III IFN suppression, since ITAMi signaling is specifically induced by tonic FcγRIIa activation by soluble IgG 50, while type I and III IFN suppression as described here was not induced by soluble IgG but essentially required IgG immune complex formation. Consequently, these findings suggest that the regulation of activating and inhibitory functions of FcRs is more intricate than previously imagined. Originally, the categorization of activating and inhibitory FcRs was made based on their role in various immune functions, in which activating receptors such as FcγRIIa signal through a so‐called ITAM in their cytoplasmic tail, while the inhibitory FcγRIIb contains an ITIM 32. Nevertheless, ITAMi signaling 47, 48, the observation that FcγRIIa (co‐)stimulation promotes the production of anti‐inflammatory cytokines such as IL‐10 17, 22, and the recent finding that FcγRIIb can induce ITIM‐independent immune activation 51 already indicated that the categorization into activating or inhibitory receptors does not necessarily reflect pro‐ and anti‐inflammatory functions in general 52. Here, our data further illustrate that the categorization into activating and inhibitory FcRs may be oversimplified, and that the immunological outcome of FcR (co‐) stimulation strongly depends on the specific gene and immune function in question.

Strikingly, inhibitory signaling by FcγRIIa was induced through a pathway that was Syk‐ and PI3K‐independent. Notably, almost all FcγRIIa‐mediated effects by myeloid cells, including cytokine induction and phagocytosis, are completely dependent on Syk and PI3K 32, 33, 34. Even aforementioned ITAMi signaling depends on (transient) activation of Syk 50, although it has not been described to be dependent on PI3K. To our knowledge, Syk‐/PI3K‐independent signaling of FcγRIIa is restricted to reports on platelet activation, in which ROS production and serotonin secretion is partially Syk‐independent 53, 54, 55. Combined, these data suggest that inhibitory signaling by IgG immune complexes is induced by signaling through a novel, non‐canonical signaling pathway of FcγRIIa. Interestingly, FcγRIIa–TLR3 crosstalk simultaneously inhibited type I and III IFN while amplifying the production of pro‐inflammatory cytokines, such as IL‐1β and TNF 22. This indicates that stimulation of FcγRIIa with IgG immune complexes activates two parallel pathways, one ‘activating’ pathway that is Syk‐/PI3K‐dependent for the amplification of pro‐inflammatory cytokines (as well as various other immunological functions such as phagocytosis), and one ‘inhibitory’ pathway that is Syk‐/PI3K‐independent that selectively suppresses type I and III IFN and ISG responses. The inhibitory pathway is likely to inhibit IFN‐β production by DCs through inhibition of expression of IRF1, which is one of the main transcription factors of type I IFNs 56, 57. This finding is in line with previous transcriptomics data that also identified IRF1 as one of the main downregulated genes upon co‐stimulation of human monocytes with complexed IgG 58. Interestingly, the signaling module in the cytoplasmic tail of FcγRIIa, characterized by an ITAM sequence, is a common signaling module that is used by a variety of receptors including the BCR, T cell receptor, and other members of the FcR family 59. Therefore, for future studies it will be very interesting to determine whether the same inhibitory signaling pathway is also induced by other ITAM‐bearing receptors, which may have important consequences for immunological responses of numerous leukocytes including B cells, T cells, NK cells, neutrophils, and mast cells.

Taken together, our data identify a novel negative feedback mechanism of type I and III IFN responses, which may contribute to an efficient transition from innate to adaptive immunity during primary viral infection. This negative feedback mechanism is dependent on inhibitory, non‐canonical signaling by FcγRIIa, which is most likely induced by recognition of IgG opsonized viruses during both late‐phase primary and secondary viral infections. From a therapeutic point of view, activation of this pathway may be a useful tool to promote antiviral immunity, or to specifically suppress type I and III IFN‐associated pathology without compromising antiviral immunity in general.

## Materials and methods

### IgG binding ELISA

Influenza A virus (H3N2) and RSV (A2) were coated overnight in coating buffer (0.05 M carbonate buffer, pH 9.6) in 96‐well high‐affinity plates (Maxisorp; Nunc). Wells were washed three times and blocked with PBS (Fresenius Kabi) containing 1% BSA (Sigma–Aldrich) for 1 h at room temperature. Wells were again washed three times and subsequently incubated with 1 mg/mL pooled IgG from healthy donors (Nanogam; Sanquin Blood Supply) or 1 mg/mL irrelevant IgG (Humira; AbbVie) for 1 h at room temperature. Wells were then washed three times with PBS containing 1% BSA and incubated with anti‐human IgG‐HRP (MH16‐1; Sanquin Blood Supply) for 30 min at room temperature. Wells were washed five times and subsequently developed using TMB substrate solution (Merck). The reaction was stopped using 1 M H_2_SO_4_ (Merck) and IgG binding was determined by analyzing absorbance at 450 nm (reference filter 655 nm) using a photospectrometer (Molecular Devices).

### Cells

PBMCs were isolated from heparinized peripheral blood from healthy donors by density gradient centrifugation on Lymphoprep (Nycomed). For DC, macrophage, or LC culture, monocytes were isolated using Percoll (Pharmacia) density gradients. Cells were cultured for 6 days in IMDM (Lonza) containing 5% fetal bovine serum (FBS; Biowest) and 86 μg/mL gentamicin (Gibco) supplemented with 20 ng/mL recombinant human GM‐CSF (Invitrogen) and 2 ng/mL recombinant human IL‐4 (Miltenyi Biotec) for DCs, 20 ng/mL recombinant human GM‐CSF for macrophages, or 20 ng/mL recombinant human GM‐CSF, 2 ng/mL recombinant human IL‐4 and 10 μg/mL recombinant human TGF‐β1 (R&D Systems) for LCs. At day 2 or 3, half of the medium was replaced by fresh medium containing cytokines. Monocytes were isolated from PBMCs by MACS isolation using CD14 microbeads (Miltenyi Biotec) for direct stimulation or analysis.

### Mouse BM‐derived DCs

Bone marrow was collected from 20‐week‐old female WT C57BL/6JR mice (experiments were approved by the animal experiments committee of the Academic Medical Center, Amsterdam). After RBC lysis, BM cells were cultured for 10 days in Tissue Culture Medium (RPMI 1640, 5% FBS, 50 μg/mL gentamicin, 0.05 mM 2‐ME) supplemented with 20 ng/mL recombinant mouse GM‐CSF (Thermo Scientific, Rockford, IL). At day 3 and 7, fresh medium containing GM‐CSF was added. At day 10, cells were harvested and stimulated, using 2 μg/mL mouse serum IgG coated in Maxisorp plates (Nunc) and 20 μg/mL Poly I:C (Sigma–Aldrich).

### Ethics statement

PBMCs used in this study derived from buffy coats obtained from healthy donors, as anonymously provided by Sanquin Bloedvoorziening, Amsterdam. Experiments using animals were carried out in accordance with the Dutch Experiment on Animals Act and approved by the Animal Care and Use Committee of the University of Amsterdam (Permit number: DRI‐102785).

### Stimulation

Cells were harvested, washed, and stimulated in 96‐well culture plates (30 000–50 000 cells/well) with influenza or RSV. Human IgG (1 mg/mL; Nanogam) was added after 30 min to opsonize viruses without causing reduced viral infection of the cells. The optimal amount of virus for IFN‐β mRNA induction by DCs was determined in a titration experiment.

To stimulate various virus‐sensing receptors, cells were stimulated with 20 μg/mL Poly I:C (Sigma–Aldrich), 1 μg/mL Poly I:C‐HMW‐LyoVec (Invivogen), and 10 μg/mL Poly (dG:dC)/ LyoVec (Invivogen). To mimic IgG complex formation, wells of 96‐well Maxisorp plates were incubated overnight with 2 μg/mL human IgG (Nanogam) and subsequently blocked with PBS containing 10% FBS. Heat aggregated immunoglobulins (HAGGs) were made by heating IgG (Sigma–Aldrich) for 1 h at 63°C and subsequent centrifugation for 10 min at 13 000*g* to separate large, insoluble HAGGs from small soluble HAGGs. Soluble HAGGS were diluted in PBS and the concentration was determined by spectrophotometry (NanoDrop: Thermo Scientific). HAGGs (100 μg/mL) were used for stimulation. BSA‐ and IgG‐coated beads were made as described previously 60. In short, CNBr‐activated Sepharose beads (GE Healthcare Life Sciences) were coupled with 3 μg purified serum IgG (Sigma–Aldrich) or BSA (Roche Diagnostics), according to the manufacturers’ instructions. IgG purity was tested by SDS electrophoresis and was ≥95%.

FcyRIIa/b were blocked by pre‐incubating DCs with 20 μg/mL of anti‐FcyRIIa (CD32a; IV.3; Stemcell Technologies) or anti‐FcyRIIb (CD32b; 2B6; MacroGenics) for 30 min at 4 degrees, after which stimuli and culture medium were added resulting in a final concentration of 5 μg/mL. For blocking TNF production, cells well treated with 10 μg/mL certolizumab. PI3K was inhibited by adding 100 nM wortmannin (Santa Cruz Biotechnology), 5 μM LY294002 (Selleckchem), or 100 nM idelalisib (Selleckchem) to the cells.

For silencing of Syk, DCs were harvested at day 3. Cells were microporated (20 ms, 1500V; Neon Transfection System; Life Technologies) in the presence of 250 nM SMARTpool Syk si‐RNA or control si‐RNA (both Darmacon), and cultured for three more days in the presence of GM‐CSF and IL‐4.

### CD8^+^ T cell stimulation and analysis

To study CD8^+^ T cell proliferation and functionality, 5000 DCs were stimulated as indicated and co‐cultured with 20 000 allogeneic naïve CD8^+^ T cells (CD8^+^, CD27^+^, CD45RO^−^, and CD45RA^+^) in the presence of 1 pg/mL *Staphylococcus aureus* enterotoxin B (SEB; Sigma–Aldrich). To determine proliferation, CD8^+^ T cells were incubated with 0.5 μM CFSE (Invitrogen) and washed extensively prior to co‐culture. At day 3 or 4, cells were incubated overnight with the modified thymidine analogue EdU (Click‐iT kit; Invitrogen) and further processed according to the manufacturer's instructions. The percentage of divided cells (EdU^+^ or CFSE^−^) was determined by flow cytometry (Canto II, BD Biosciences). To determine intracellular granzyme B expression, cells were harvested at day 4 or 5, washed with PBS, fixated with 4% formaldehyde (Sigma–Aldrich) for 15 min, washed again, permeabilized with 0.5% saponin (Calbiochem) in PBS containing 0.5% BSA (PAA) and 0.1% sodium azide (Merck), and stained with anti‐granzyme B‐PE (Sanquin Blood Supply) and analyzed by flow cytometry. For intracellular IFN‐γ or TNF staining, CD8^+^ T cells were restimulated at day 4 or 5 with 100 ng/mL PMA, 1 μg/mL ionomycin, and 10 μg/mL brefeldin A (all Sigma–Aldrich) for 6 h, washed, fixated, and permeabilized as described above, stained with anti‐IFN‐y‐ FITC and anti‐TNF‐APC (both BD Biosciences) and analyzed by flow cytometry.

### Enzyme linked immunosorbent assay

For analysis of cytokine production, supernatants were harvested after overnight stimulation and stored at –20ᵒC. IFN‐β and CXCL10 cytokine production after stimulation with Poly I:C was determined by harvesting the supernatants 3 h after stimulation and supernatants were again stored at –20ᵒC. Cytokine levels in supernatants were measured by ELISA, using an IFN‐β ELISA kit (PBL Assay Science), antibody pairs for CXCL10 (R&D Systems), TNF (MAb1; MAb11; eBioscience), and IL‐1β (CT213‐c; CD2013‐d; U‐CyTech).

### Quantitative RT‐PCR

For mRNA‐level analysis the cells were lysed at the indicated time points, after which mRNA was extracted using the RNeasy Mini Kit (Qiagen) and cDNA was synthesized using the RevertAid H Minus First Strand cDNA Synthesis Kit (Thermo Scientific). Quantitative RT‐PCR was performed on StepOnePlus^TM^ Real‐Time PCR System (Applied Biosystems) using TaqMan gene expression assays for IFN‐β (Hs01077958_s1), IFN‐λ1 (Hs00601677_g1), CXCL10 (Hs00171042_m1), TNF (Hs00174128_m1), Syk (Hs00895377_m1), IRF1 (Hs00971965_m1), IRF3 (Hs01547283_m1), IRF7 (Hs00185375_m1), and GAPDH (4310884E) according to the protocol of the manufacturer (ThermoFisher). Other mRNA levels were determined by using SYBR green (Applied Biosystems) and primer pairs as listed in Tables 1 and 2. mRNA levels were normalized to the *Ct*‐values of the housekeeping gene GAPDH and fold change values were calculated compared with an unstimulated control sample (*t* = 0 h).

**Table 1:** Primers for quantitative RT‐PCR (human)

Target mRNA	Forward primer (5′–3′)	Reverse primer (5′–3′)
**APOBEC3G**	TTGAGCCTTGGAATAATCTGCC	TCGAGTGTCTGAGAATCTCCCC
**CD70**	ATCACACAGGACCTCAGCA	CACCTGGATGTGTACCATGT
**EBI3**	CGTGCCTTTCATAACAGAGCA	GACGTAGTACCTGGCTCGG
**GAPDH**	GAAGGTGAAGGTCGGAGTC	GAAGATGGTGATGGGATTT
**IL12A**	ACCGCTTTGCGGAATCTCA	CTGAAGCGTGGTGGAGATGAA
**IL27**	ATCTCACCTGCCAGGAGTGAAC	TGAAGCGTGGTGGAGATGAA
**Influenza A**	GACAAGACCAATCCTGTCACYTCTG	AAGCGTCTACGCTGCAGTCC
**MX1**	CTGAAGGAGCGGCTTGCACGG	CCACGGCTAACGGATAAGCAGGAA
**OAS2**	CCAGCTGAGAGCAATGGGAA	AGGACAAGGGTACCATCGGA
**RSV**	ATGAACAGTTTAACATTACCAAGT	GTTTTGCCATAGCATGACAC
**STAT1**	GCGCGCAGAAAAGTTTCATTT	GAGACATCCTGCCACCTTGT

**Table 2:** Primers for quantitative RT‐PCR (mouse)

Target mRNA	Forward primer (5′–3′)	Reverse primer (5′–3′)
**IFN‐β**	AAGAGTTACACTGCCTTTGCCATC	CACTGTCTGCTGGTGGAGTTCATC
**CXCL10**	GACGGTCCGCTGCAACTG	GCTTCCCTATGGCCCTCATT
**GAPDH**	ACTCCCACTCTTCCACCTTC	CACCACCCTGTTGCTGTAG

### Data analysis

Data were analyzed for statistical significance using paired two‐tailed Student's *t*‐test with GraphPad Prism version 5.01 software (GraphPad Software).

### Data availability

All relevant data is contained within the manuscript and is available from the authors upon reasonable request.

## Author contributions

M.N., W.H., L.T.C.V., J.A.v.B., E.W.M.T.K., and J.d.D. performed research. M.N., W.H., L.T.C.V., M.L.K., D.LP.B., J.d.D., and E.C.d.J. designed research and analyzed data. M.H.H., D.E., T.W.K., and T.B. contributed reagents or materials. J.d.D. devised concept. M.N., L.T.C.V., and J.d.D. wrote the manuscript. T.W.K., M.v.E., D.L.P.B., and E.C.d.J. reviewed and edited the manuscript.

## Conflict of interest

D.B. is a part‐time employee of Union Chimique Belge. T.B. is a full‐time employee of AIMM Therapeutics. The other authors have no financial conflicts of interest.

**Abbreviations**
c‐IgG: complexed‐IgG
CDS: cytosolic DNA sensor
ISG: IFN‐stimulated gene
RLR: RIG‐I‐like receptor
STING: Stimulator of Interferon Genes

## Supporting information

Peer review correspondence

**Figure S1. Co‐stimulation of human DCs**. DCs were stimulated with Poly I:C, c‐IgG, or the combination (A,B). mRNA expression (at indicated time points) was determined by quantitative RTPCR. Each pair of dots represents one donor, representative of twenty independent experiments. *p < 0.05, **p < 0.01, ***p < 0.001, paired two‐tailed Student's *t*‐test. IFN‐β and IFN‐λ1 mRNA levels were compared at t=3h and CXCL10 mRNA levels were compared at t=6h (A). Data shown are from one experiment, representative of three independent experiments (B).
**Figure S2. TNF induction**. DCs were stimulated with Poly I:C, either or not in combination with c‐IgG (A,B). Cytokine levels were determined 3 and 6 h after stimulation by ELISA, mean ± SEM of triplicate (A). mRNA expression (at indicated time points) was determined by quantitative RT‐PCR. Data shown are from one experiment, representative of three independent experiments (A,B).
**Figure S3. Syk silencing**. Syk in human DCs was silenced using specific si‐RNA. (A) Syk mRNA expression of unstimulated DCs after Syk silencing (si‐Syk) or non‐targeted control silencing (si‐C). (B) 24 h after stimulation, cytokine levels were determined by ELISA, mean+SEM of triplicate. (A and B) Data shown are from one experiment, representative of three independent experiments.
**Figure S4. PI3K inhibitors**. DCs were stimulated with Poly I:C alone or in combination with c‐IgG (A,B). PI3K was inhibited by LY294002 (A) or idelalisib (B). mRNA expression (at indicated time points) was determined by quantitative RT‐PCR. Data shown are from one experiment, representative of three independent experiments.
