# Supplementary material for: Fc gamma receptor IIa suppresses type I and III interferon production by human myeloid immune cells
Source: Eur J Immunol. 2018 Sep 14;48(11):1796–809. doi: 10.1002/eji.201847615 (PMC6282563; doi:10.1002/eji.201847615)
Supplement: Supplementary file 2 — Figure S1. Co‐stimulation of human DCs. DCs were stimulated with Poly I:C, c‐IgG, or the combination (A,B). mRNA expression (at indicated time points) was determined by quantitative RTPCR. Each pair of dots represents one donor, representative of twenty independent experiments. *p < 0.05, **p < 0.01, ***p < 0.001, paired two‐tailed Student's t‐test. IFN‐β and IFN‐λ1 mRNA levels were compared at t=3h and CXCL10 mRNA levels were compared at t=6h (A). Data shown are from one experiment, representative of three independent experiments (B). Figure S2. TNF induction. DCs were stimulated with Poly I:C, either or not in combination with c‐IgG (A,B). Cytokine levels were determined 3 and 6 h after stimulation by ELISA, mean ± SEM of triplicate (A). mRNA expression (at indicated time points) was determined by quantitative RT‐PCR. Data shown are from one experiment, representative of three independent experiments (A,B). Figure S3. Syk silencing. Syk in human DCs was silenced using specific si‐RNA. (A) Syk mRNA expression of unstimulated DCs after Syk silencing (si‐Syk) or non‐targeted control silencing (si‐C). (B) 24 h after stimulation, cytokine levels were determined by ELISA, mean+SEM of triplicate. (A and B) Data shown are from one experiment, representative of three independent experiments. Figure S4. PI3K inhibitors. DCs were stimulated with Poly I:C alone or in combination with c‐IgG (A,B). PI3K was inhibited by LY294002 (A) or idelalisib (B). mRNA expression (at indicated time points) was determined by quantitative RT‐PCR. Data shown are from one experiment, representative of three independent experiments. [file EJI-48-1796-s002.pdf]

Supplemental Figures

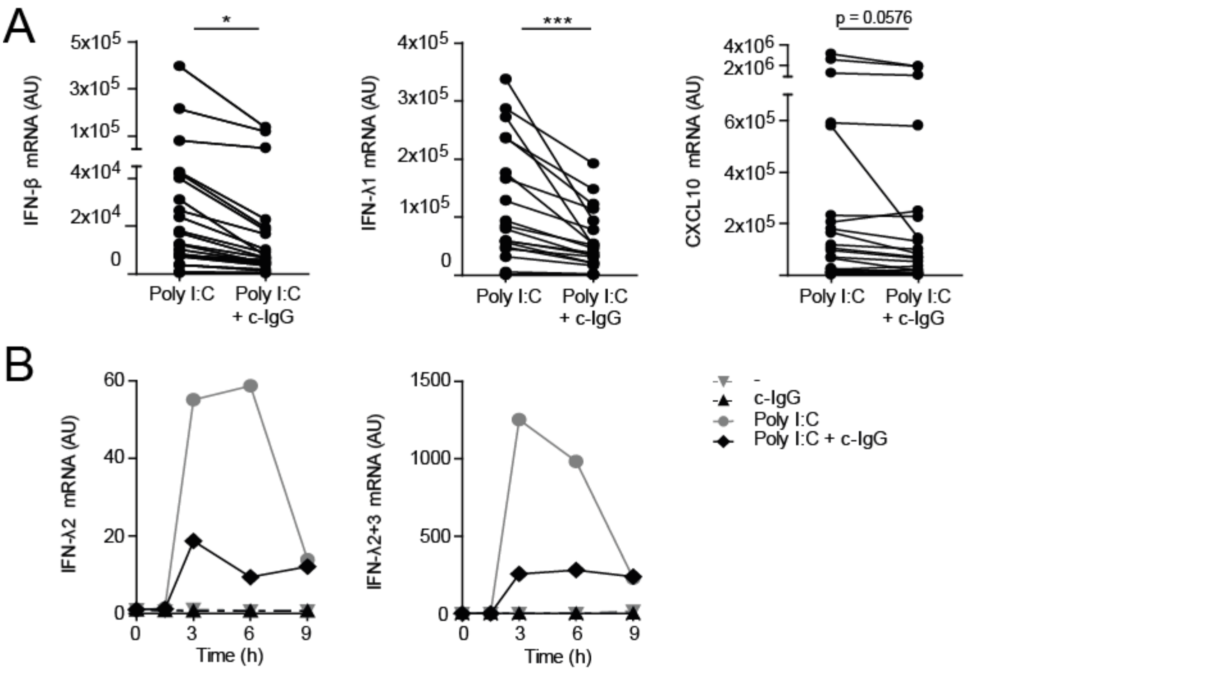

Figure S1. Co-stimulation of human DCs. DCs were stimulated with Poly I:C, c-IgG, or the combination (A,B). mRNA expression (at indicated time points) was determined by quantitative RT-PCR. Each pair of dots represents one donor, representative of twenty independent experiments. *$P < 0.05$, **$P < 0.01$, ***$P < 0.001$, paired two-tailed Student's *t*-test. IFN-β and IFN-λ1 mRNA levels were compared at t=3h and CXCL10 mRNA levels were compared at t=6h (A). Data shown are from one experiment, representative of three independent experiments (B).

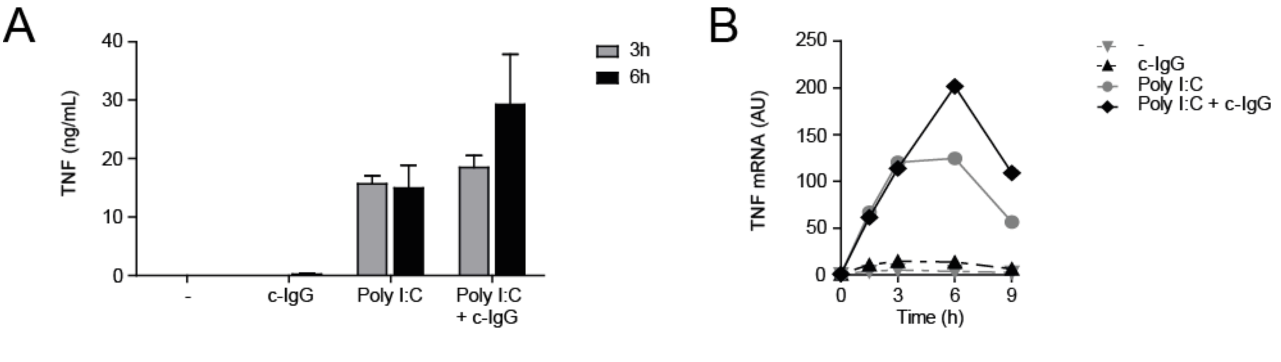

Figure S2. TNF induction. DCs were stimulated with Poly I:C, either or not in combination with c-IgG (A,B). Cytokine levels were determined 3h and 6h after stimulation by ELISA, mean+SEM of triplicate (A). mRNA expression (at indicated time points) was determined by quantitative RT-PCR. Data shown are from one experiment, representative of three independent experiments (A,B).

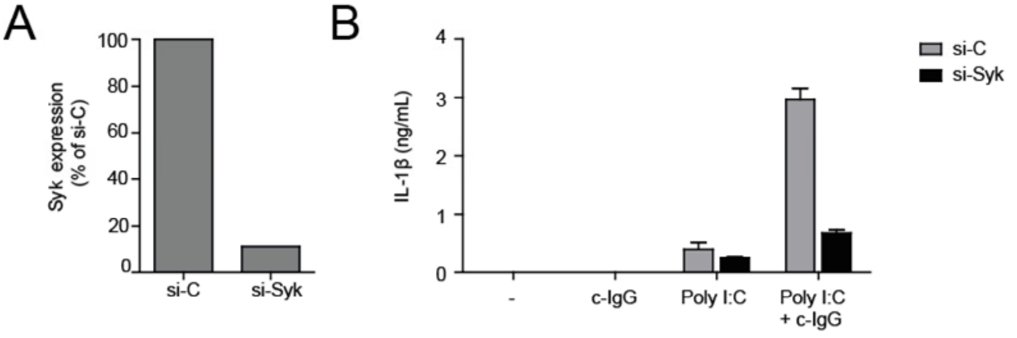

Figure S3. Syk silencing. Syk in human DCs was silenced using specific si-RNA. (A) Syk mRNA expression of unstimulated DCs after Syk silencing (si-Syk) or non-targeted control silencing (si-C). (B) 24h after stimulation, cytokine levels were determined by ELISA, mean+SEM of triplicate. (A-B) Data shown are from one experiment, representative of three independent experiments.

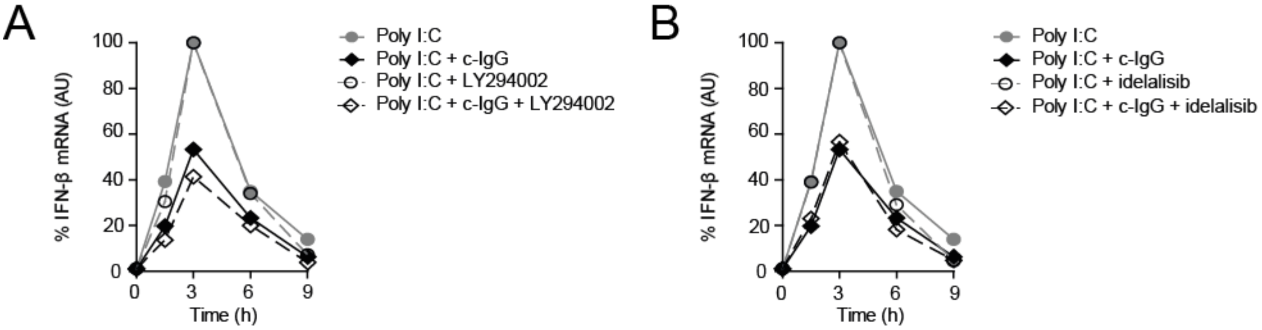

Figure S4. PI3K inhibitors. DCs were stimulated with Poly I:C alone or in combination with c-IgG (A,B). PI3K was inhibited by LY294002 (A) or idelalisib (B). mRNA expression (at indicated time points) was determined by quantitative RT-PCR. Data shown are from one experiment, representative of three independent experiments.
