# Supplementary material for: Fc gamma receptor IIa suppresses type I and III interferon production by human myeloid immune cells
Source: Eur J Immunol. 2018 Sep 14;48(11):1796–809. doi: 10.1002/eji.201847615 (PMC6282563; doi:10.1002/eji.201847615)
Supplement: Supplementary file 1 — Peer review correspondence [file EJI-48-1796-s001.pdf]

European Journal of Immunology

Fc gamma receptor IIa suppresses type I and III interferon production by human myeloid immune cells

Newling, Melissa; Hoepel, Willianne; Vogelpoel, Lisa; Heineke, Marieke; van Burgsteden, Johan; Taanman-Kueter, Esther; Eggink, Dirk; Kuijpers, Taco; Beaumont, Tim; van Egmond, Marjolein; Kapsenberg, Martien; Baeten, Dominique; den Dunnen, Jeroen; de Jong, Esther C.

Correspondence: Jeroen den Dunnen, Amsterdam Rheumatology and Immunology Center, location Academic Medical Center (AMC), Amsterdam, The Netherlands

---

Review Timeline:	Submission date:	23-Mar-2018
	First Editorial decision:	25-Apr-2018
	Revision/s received:	17-Jul-2018
	Accepted:	3-Sep-2018

Handling Executive Committee member: Prof. Annette Oxenius

Please note that the correspondence below does not include the standard editorial instructions regarding preparation and submission of revised manuscripts, only the scientific revisions requested and addressed.

First Editorial Decision - 25-Apr-2018

Dear Mrs. Newling,

Please accept our sincere apologies for the delay in the decision, there was a delay in receiving one of the referees' reports.

Manuscript ID eji.201847615 entitled "IgG opsonization of viruses suppresses type I and III interferon production by human myeloid antigen-presenting cells via inhibitory signaling by Fc gamma receptor IIa" which you submitted to the European Journal of Immunology has been reviewed. The comments of the referees are included at the bottom of this letter.

Referees found the study interesting. However, they both think that additional investigation is needed to support the conclusions.

A revised version of your manuscript that takes into account the comments of the referees will be reconsidered for publication. Should you disagree with any of the referees' concerns, you should address this in your point-by-point response and provide solid scientific reasons for why you will not make the requested changes.

You should also pay close attention to the editorial comments included below. **In particular, please edit your figure legends to follow Journal standards as outlined in the editorial comments. For all data, including the new data generated for the revision of the manuscript, please report in the Figure legends the number of independent experiments and number of samples per experiment (or experimental replicates). For flow cytometry data please show the full gating strategy, including the percentage of cells in the region or gate or event count. In the histograms/dot plots shown please report which fluorochromes were used and the scaling in the axis (log/lin). Failure to do this will result in delays in the re-review process.**

Please note that submitting a revision of your manuscript does not guarantee eventual acceptance, and that your revision will be re-reviewed by the referees before a decision is rendered.

If the revision of the paper is expected to take more than three months, please inform the editorial office. Revisions taking longer than six months may be assessed by new referees to ensure the relevance and timeliness of the data.

Once again, thank you for submitting your manuscript to European Journal of Immunology and we look forward to receiving your revision.

Yours sincerely,
Marta Vuerich

On behalf of
Prof. Annette Oxenius

Dr. Marta Vuerich
Editorial Office
European Journal of Immunology
e-mail: ejied@wiley.com
www.eji-journal.eu

****

European Journal of Immunology

Reviewer: 1

Comments to the Author
Manuscript Nr.: eji.201847615
Newling et al., "IgG opsonization of viruses suppresses type I and III interferon production by human myeloid antigen-presenting cells via inhibitory signaling by Fc gamma receptor IIa"

The authors demonstrate that immune complexes, including opsonized influenza and respiratory syncytial viruses, attenuate type I and III interferon (IFN) production by human monocyte-derived myeloid DCs. This affects also IFN stimulated gene (ISG) expression, but not production of IL-12 and IL-27 or co-stimulatory molecule up-regulation like CD70. The authors identify FcgammaRIIa as the responsible receptor, but the activating signaling by this receptor does not seem to be involved in this type I and III IFN production inhibition. From these data the authors speculate that immune complexes terminate anti-viral IFN production after viral infection via novel FcgammaRIIa signaling.

The reported findings could be potentially interesting, if the authors can clarify that viral infection is unaltered under their experimental conditions, how their monocyte-derived DCs detect CpG and give some indication on the FcgammaRIIa signaling that is involved in the inhibition of IFN production.

Major comments:

1. In order to exclude that opsonization blocks infection and thereby prevents all cytosolic RNA detection in response to RNA virus infection, the authors have to demonstrate that infection is similar between virus exposure with and without antibody addition, for example for the reported conditions after adding antibodies 30 minutes after infection.
2. TLR9 is thought to be primarily expressed by plasmacytoid DCs. How do the authors envision that CpG induces type I and III interferon in myeloid DCs, which they then report to be attenuated by immune complexes? Do they authors detect expression of TLR9 by human monocyte-derived DCs?
3. How does FcgammaRIIa inhibit type I and III interferon production? Some information how an activating Fcgamma receptor can achieve this, should be provided.

Minor comments:

1. Most datasets are shown as representative experiments without statistical evaluation. The authors should either try to summarize their data in additional figures with statistical evaluation or report statistical significance at least in the text.

In summary, without some indication how the activating FcgammaRIIa receptor inhibits type I and III IFN

production, the reported findings seem rather preliminary.

Reviewer: 2

Comments to the Author

The study by Newling and colleagues studies how opsonized viruses influence type I interferon responses in vitro. By using largely in vitro assays with human blood derived dendritic cells the authors demonstrate that opsonized virus particles inhibit type I interferon responses. The authors suggest that an as yet undefined inhibitory signaling event triggered through IC binding to FcRIIa may be responsible for this inhibitory effect. While the results are presented clearly, some major issues with the study design and most forward the interpretation of the results lower my enthusiasm for the study at present.

While the authors show that adding IgG 0.5 hrs after virus does not alter virus mRNA in the cell they do not show proof that a virus IC is generated under these conditions. The could elute virus IgG complexes from the cell surface to proof this.

Most forward, I have a major concern with the results in Figures 2-6 as the authors never use immune complexes but instead immobilize IgG on plastic. This by no means allows inferring how a soluble immune complex would activate a DC and cannot be compared to an opsonized virus. There are multiple possibilities to generate bona fide human immune complexes and without this, the overall conclusions of the manuscript are flawed.

Further along these lines, the vast majority of the data has not been generated with virus ICs. To maintain the title of the paper it would be critical to show that FcRIIa blockade enhances type I IFN production in the context of opsonized virus instead of just using TLR ligands.

Furthermore, the authors state that human DCs behave different than mouse DCs. This statement (and the shown data) is problematic as human DCs and mouse DCs were generated with different protocols (blood versus bone marrow, IL4 present in human DC cultures versus absent in mouse DC cultures, etc.). To make this statement the authors should generate DCs under the same conditions. Moreover, DCs exist in many subsets, which may or may not react in the way as shown for blood derived DCs. This should be discussed in much greater detail.

European Journal of Immunology

First Revision – authors' response -17-Jul-2018

We thank the reviewers for their comments and suggestions and are glad to hear that they consider this work of potential interest for publication in the European Journal of Immunology. Below we have made a point-by-point response to the reviewer's comments.

Reviewer 1

The reported findings could be potentially interesting, if the authors can clarify that viral infection is unaltered under their experimental conditions, how their monocyte-derived DCs detect CpG and give some indication on the FcgammaRIIa signaling that is involved in the inhibition of IFN production.

Major comments:

1. In order to exclude that opsonization blocks infection and thereby prevents all cytosolic RNA detection in response to RNA virus infection, the authors have to demonstrate that infection is similar between virus exposure with and without antibody addition, for example for the reported conditions after adding antibodies 30 minutes after infection.

The reviewer is right that it should be verified that the infection rate is similar between virus exposure with and without antibodies. We now show this in Figure 1B (previously, this was shown as supplementary data, but we realize that this important control should be included as a main figure).

2. TLR9 is thought to be primarily expressed by plasmacytoid DCs. How do the authors envision that CpG induces type I and III interferon in myeloid DCs, which they then report to be attenuated by immune complexes? Do they authors detect expression of TLR9 by human monocyte-derived DCs?

The reviewer certainly raises a good point here. We have stimulated monocyte-derived DCs (moDCs) with CpG since it has previously been described that monocyte-derived DCs express TLR9 and respond to CpG [Hoene et al. 2006 J Leukoc Biol]. Yet, a recent study identified that CpG-induced interferon production detected in moDC cultures may actually result from contamination of the cultures with small percentages of B cells, which express TLR9 [Sprokholt et al. 2017 PLOS ONE].

Since the issue of TLR9 expression by moDCs is still unclear, and also since we have merely used TLR9 as one of many examples of IFN-inducing receptors that are inhibited by FcγRIIa stimulation, we have decided to remove the CpG data from the manuscript. Please note that the data in Figure 2 still clearly demonstrates that IgG immune complexes inhibit type I IFN production by various receptors, including TLRs, RIG-I/MDA5, and cytosolic DNA sensors.

3. How does FcgammaRIIa inhibit type I and III interferon production? Some information how an activating Fcgamma receptor can achieve this, should be provided.

As requested by the reviewer, we have further looked into the mechanism of FcγRIIa-mediated type I IFN suppression. Since we identified that FcγRIIa inhibits type I IFN production at the level of gene transcription, we have assessed the expression of the main transcription factors of type I IFN, i.e. IRF1, IRF3, and IRF7. Importantly, while we did not observe any difference in IRF3 and IRF7 expression, we

European Journal of Immunology

identified that FcγRIIa co-stimulation down-regulates the expression of IRF1 (Figure 6D). This finding nicely correlates with very recent transcriptomics data that also identified IRF1 as one of the main down-regulated genes upon co-stimulation of human monocytes with complexed IgG [Zhong et al. 2018 JI]. We have also implemented this important finding in the Discussion of the manuscript (p. 15 lines 336-340).

Minor comments:

1. Most datasets are shown as representative experiments without statistical evaluation. The authors should either try to summarize their data in additional figures with statistical evaluation or report statistical significance at least in the text.

As requested, we have now added statistical evaluation for several key experiments. These are depicted in Figures 1D, 1F, 2B, and Supplementary Figure 1A.

Reviewer 2

While the authors show that adding IgG 0.5 hrs after virus does not alter virus mRNA in the cell they do not show proof that a virus IC is generated under these conditions. The could elute virus IgG complexes from the cell surface to proof this.

The reviewer is right that it is important to verify that the antibodies indeed recognize and bind to the viruses. Therefore, we have assessed binding of antibodies to both influenza and RSV using an ELISA setup (Figure 1A), which confirmed IgG binding to the viruses (previously, this was shown as supplementary data, but we agree that this control should be included as a main figure).

Most forward, I have a major concern with the results in Figures 2-6 as the authors never use immune complexes but instead immobilize IgG on plastic. This by no means allows inferring how a soluble immune complex would activate a DC and cannot be compared to an opsonized virus. There are multiple possibilities to generate bona fide human immune complexes and without this, the overall conclusions of the manuscript are flawed.

Another good point by the reviewer. To really make sure that soluble immune complexes induce the same responses as plate-bound IgG we have tested this not in one, but in two different ways. First we have co-stimulated our cells with the soluble fraction of heat-aggregated immunoglobulins (also known as “haggs”). Second, we have co-stimulated our cells with IgG-coated beads, which we have compared with control (BSA-coated) beads. Importantly, both soluble IgG immune complexes yielded the exact same results as plate-bound IgG and IgG opsonized viruses (Figure 2D and 2E), demonstrating that plate-bound and soluble IgG immune complexes indeed suppress type I IFN production by human DCs in a similar manner.

Further along these lines, the vast majority of the data has not been generated with virus ICs. To maintain the title of the paper it would be critical to show that FcRIIa blockade enhances type I IFN production in the context of opsonized virus instead of just using TLR ligands.

European Journal of Immunology

Because of a number of technical problems we were not able to perform the FcγRIIa blockade experiment before the resubmission deadline. Therefore, following the reviewer's suggestion, we have adjusted the title of the manuscript so it now no longer includes viruses (new title: "Inhibitory signaling by Fc gamma receptor IIa suppresses type I and III interferon production by human myeloid immune cells").

Furthermore, the authors state that human DCs behave different than mouse DCs. This statement (and the shown data) is problematic as human DCs and mouse DCs were generated with different protocols (blood versus bone marrow, IL4 present in human DC cultures versus absent in mouse DC cultures, etc.). To make this statement the authors should generate DCs under the same conditions. Moreover, DCs exist in many subsets, which may or may not react in the way as shown for blood derived DCs. This should be discussed in much greater detail.

We have indeed used different protocols for the generation of human and mouse DCs. The main issue here are the relatively large inter-species differences, which requires the use of different reagents for the *in vitro* differentiation of precursor cells into DCs (the same hold true for e.g. *in vitro* differentiated macrophage subsets, which are generated in different ways for mouse and man). We have chosen to use the most commonly used DC differentiation protocol for both human and mouse, for optimal relevance and comparison regarding previous and future studies. Yet, since the reviewer is right that these differentiation protocols differ, we have removed or attenuated our statements regarding human versus mouse differences regarding IFN-β suppression in Abstract (p. 3 lines 42-43), Results (p. 9 lines 182-183), and Discussion (p. 13-14 lines 285-290). Furthermore, we agree with the reviewer's point that not all DCs are the same, which as requested we have added to the Discussion of the manuscript (p. 13 lines 267-273).

Taken together, we believe that the additional experimental data and textual adjustments have greatly strengthened the manuscript. We thank the reviewers for their comments and suggestions and look forward to their reply.

Kind regards,

Jeroen den Dunnen (also on behalf of all co-authors)

Second Editorial Decision - 20-Aug-2018

Dear Mrs. Newling,

It is a pleasure to provisionally accept your manuscript entitled "Inhibitory signaling by Fc gamma receptor IIa suppresses type I and III interferon production by human myeloid immune cells" for publication in the European Journal of Immunology. For final acceptance, please follow the instructions below and return the requested items as soon as possible as we cannot process your manuscript further until all items listed below are dealt with.

Please note that EJI articles are now published online a few days after final acceptance (see Accepted Articles: https://onlinelibrary.wiley.com/toc/15214141/0/ja). The files used for the Accepted Articles are the final files and information supplied by you in Manuscript Central. You should therefore check that all the information (including author names) is correct as changes will NOT be permitted until the proofs stage.

We look forward to hearing from you and thank you for submitting your manuscript to the European Journal of Immunology.

Yours sincerely,
Laura Soto Vazquez

on behalf of
Prof. Annette Oxenius

Mrs. Laura Soto Vazquez
Editorial Office
European Journal of Immunology
e-mail: ejied@wiley.com
www.eji-journal.eu
